# Soft Tissue Sarcoma of Lower Extremity: Functional Outcome and Quality of Life

**DOI:** 10.1245/s10434-021-09774-6

**Published:** 2021-03-19

**Authors:** Gilber Kask, Jussi P. Repo, Erkki J. Tukiainen, Carl Blomqvist, Ian Barner-Rasmussen

**Affiliations:** 1 Department of Plastic Surgery, Helsinki University Hospital and University of Helsinki, HUS, Helsinki, Finland; 2 Tampere University Hospital, Department of Orthopaedics and Traumatology, Unit of Musculoskeletal Surgery, Tampere, Finland; 3 Department of Orthopaedics and Traumatology, Helsinki University Hospital and University of Helsinki, Helsinki, Finland; 4 Comprehensive Cancer Center, Helsinki University Hospital and University of Helsinki, HUS, Helsinki, Finland; 5 Department of Oncology, Örebro University Hospital, Örebro, Sweden

## Abstract

**Background:**

Few studies have focused on patient-related factors in analyzing long-term functional outcome and health-related quality of life (HRQoL) in patients with postoperative lower extremity soft tissue sarcoma (STS).

**Objective:**

The purpose of this study was to investigate factors associated with postoperative functional outcome and HRQoL in patients with lower extremity STS.

**Methods:**

This cross-sectional study was performed in a tertiary referral center using the Toronto Extremity Salvage Score (TESS), Quality-of-Life Questionnaire (QLQ)-C30 and 15 Dimension (15D) measures. Functional outcome and HRQoL data were collected prospectively. All patients were treated by a multidisciplinary team according to a written treatment protocol.

**Results:**

A total of 141 patients who had undergone limb-salvage surgery were included. Depending on the outcome measure used, 19–51% of patients were completely asymptomatic and 13–14% of patients had an unimpaired HRQoL. The mean score for TESS, 15D mobility score, and QLQ-C30 Physical Functioning scale were 86, 0.83, and 75, respectively, while the mean score for 15D was 0.88, and 73 for QLQ-C30 QoL. Lower functional outcome was statistically significantly associated with higher age, higher body mass index (BMI), and the need for reconstructive surgery and radiotherapy, while lower HRQoL was statistically significantly associated with higher age, higher BMI, and reconstructive surgery.

**Conclusion:**

Functional outcome and HRQoL were generally high in this cross-sectional study of patients with STS in the lower extremity. Both tumor- and treatment-related factors had an impact but patient-related factors such as age and BMI were the major determinants of both functional outcome and HRQoL.

**Supplementary Information:**

The online version of this article (10.1245/s10434-021-09774-6) contains supplementary material, which is available to authorized users.

Soft tissue sarcomas (STSs) are rare heterogeneous mesenchymal tumors, representing approximately 1% of all solid malignancies in adults.^1^ More than half of STSs arise in the extremities.^2^

The aim of extremity STS treatment is patient survival and limb salvage with the best possible functional outcome. Several studies have been published on functional outcome after the treatment of patients with lower extremity STS,^3^ but fewer on long-term health-related quality of life (HRQoL).^4^–^7^ Few studies have focused on patient-related factors in analyzing long-term functional outcome and HRQoL in patients with postoperative lower extremity STS.^5^,^8^

This study provides knowledge about the expected postoperative long-term functional outcome and HRQoL in patients with lower extremity STS treated with limb-salvage surgery at a large, tertiary referral center.

The aims of this study were to document the functional outcome and HRQoL after treatment of STS in the lower extremity, and to investigate sociodemographic, oncological, and surgical factors predictive for inferior outcome.

## Methods

### Study Design

The present cross-sectional study was approved by the Ethics Committee of Helsinki University Hospital, Finland. Inclusion criteria are presented in Table 1. Suitable patients were identified from hospital databases using International Classification of Diseases, Tenth Revision (ICD-10) and NOMESCO Classification of Surgical Procedures codes. Demographic, clinical, surgical, and oncological data were collected retrospectively, whereas functional and HRQoL outcome data were obtained prospectively. Patients were asked to participate by mail.

**Table 1:** Inclusion criteria

Age 18 or above
Local disease at the time of diagnosis
Surgical treatment for lower extremity STS
Treated at Helsinki University Hospital by STS group
Treated between 2006 and 2015
Minimum postoperative follow-up 6 months
Returned signed informed consent form

### Outcome Measures

#### Toronto Extremity Salvage Score (TESS)

The Toronto Extremity Salvage Score (TESS) is the most widely used tool for outcome assessment of lower limb sarcoma.^9^–^13^ It is a self-administered patient-reported outcome measure (PROM) that includes 30 items regarding activity limitations in daily life, such as restrictions in body movement, mobility, self-care, and performance of daily tasks and routine. The raw score was converted to a score ranging from 0 to 100 points, with higher scores indicating less functional limitations.^9^

#### European Organisation for Research and Treatment of Cancer (EORTC) Quality-of-Life Questionnaire (QLQ)-C30

The European Organisation for Research and Treatment of Cancer (EORTC) Quality-of-Life Questionnaire (QLQ)-C30 is a cancer-specific, patient-reported HRQoL instrument^14^ that has been validated and shown to provide reliable measures.^15^ The QLQ-C30 consists of nine multi-item scales, including five functional scales, three symptom scales, a Global Health Status/QoL scale, and six single-item symptom measures. Scales are scored from 0 to 100. In the functional, global health, and quality-of-life scales, higher scores represent better results, while in the symptom scales, higher scores indicate more symptoms.

## Dimension (15D)

The 15 Dimension (15D) is a generic, self-administered HRQoL instrument.^16^ The questionnaire contains 15 dimensions of health: mobility, vision, hearing, breathing, sleeping, eating, speech, excretion, usual activities, mental function, discomfort and symptoms, depression, distress, vitality, and sexual activity. The 15D can be presented as a profile or as a single index score measure. The score varies between 0, representing the worst result, and 1, representing the best result.

### Used Measures

Functional outcome was measured using the TESS, QLQ-C30 Physical Functioning (PF) scale, and the 15D mobility item, while HRQoL was measured using the QLQ-C30 Global Health Status/QoL scale (QLQ-C30 QoL), and the overall score of the 15D questionnaire (15D score). For better comparison with other results, the 15D score and 15D mobility item are presented as 0, representing the worst result, and 100, representing the best possible result, in the tables displaying results of univariate and multivariate analyses. The minimal clinically important difference has been defined as ≥ 4–10 for the TESS,^17^ as ≥ 1.5 for 15D score^18^ and as ≥ 5–10 for QLQ-C30 score.^19^

### Helsinki Soft Tissue Sarcoma Group Protocol for Diagnostic Work-Up, Treatment, and Follow-Up

The Helsinki University Hospital STS group is a multidisciplinary team of plastic surgeons, oncologists, pathologists, and radiologists. The treatment of each patient is planned in weekly meetings. Magnetic resonance imaging (MRI) is performed to assess soft tissue tumors that are suspicious of sarcoma on ultrasound (US) examination in the referring institution, or clinically in our outpatient clinic. A soft tissue tumor radiologist assesses the MRI immediately after the imaging, and US-guided core-needle or, more seldom, fine-needle biopsy is performed during the same visit if a suspicion of malignancy arises. If a diagnosis of sarcoma is confirmed by our soft tissue tumor pathologist, systemic status of the disease is further examined using computed tomography (CT) of the lungs. For tumors previously biopsied or operated on outside Helsinki University Hospital, our pathologist re-evaluates all specimens. After tumor resection, the pathologist measures tumor size before sample fixation, and final histopathological examination is then carried out.

STS malignancy grading is based on a four-tiered grading system used by the Scandinavian Sarcoma Group.^20^^,^^21^ Grades 1–2 are considered low malignancy grade and grades 3–4 are considered high-grade. Wide microscopical margins are defined as 25 mm of healthy tissue or an intact fascial barrier separating the tumor from the excision margin, otherwise margins were classified as marginal or intralesional depending on whether tumor cells were present on the specimen border.^22^ After intralesional margins, re-excision is recommended in patients treated with curative intent if feasible. Wide surgical margins are recommended except for grade 1 liposarcomas, which are managed by marginal resection. After marginal or intralesional excision, external beam radiotherapy is delivered to the operative field at a dose of 50–70 Gy over a 5-week period. Selected patients with a high risk of metastatic spread receive adjuvant chemotherapy. Follow-up time is 5 years for patients with high-grade STS and 10 years for low-grade tumors.

### Clinical and Tumor Data Classification

Overweight and obesity were defined as a body mass index (BMI) ≥ 25 kg/m^2^ and ≥ 30 kg/m^2^, respectively, according to the WHO criteria.^23^ Tumor location in the lower extremity was defined as a tumor distal to the inguinal ligament anteriorly or gluteal sulcus posteriorly. Tumor depth was based on the tumor relationship to the deep fascia; tumors were defined as superficial when superficial to, and not infiltrating, the deep fascia of the limb, or otherwise were defined as deep. Complications were classified as minor and major—major when surgical re-intervention was needed. Wound closure was classified as direct wound closure, wound closure using split-thickness skin graft (STSG), or reconstructive wound closure using pedicled or microvascular flaps.

### Statistical Analysis

Descriptive statistics are presented as means and standard deviations (SDs) or counts and percentages. Follow-up was calculated from the date of the last sarcoma surgery to the date of completing the questionnaires. Univariate regression analysis was performed by comparing the results obtained from questionnaires with clinical and oncological factors. A multivariate regression model and linear regression analysis was used to examine the associations between functional outcome and HRQoL, as well as potential demographic, oncological, and clinical correlates. A *p* value < 0.05 was considered statistically significant. For statistical analysis, SPSS Statistics 24.0 software (IBM Corporation, Armonk, NY, USA) was used.

## Results

**Fig. 1:**
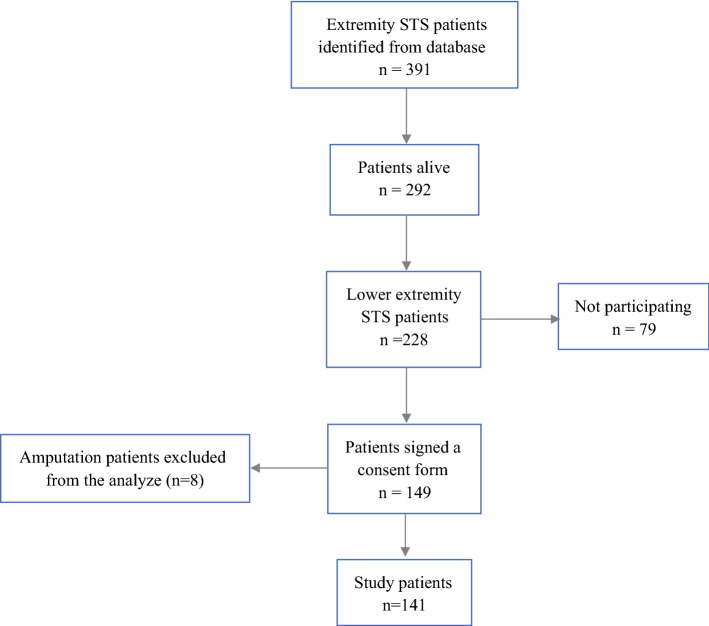
Flow of patient recruitment. *STS* soft tissue sarcoma

A total of 141 patients who had undergone limb-salvage surgery were included. Patient recruitment is described in the flow diagram shown in Fig. 1, and patient, surgical, and oncological characteristics are summarized in Table 2. Liposarcoma was the most common histological subtype, and 31 of these were grade 1. The reconstructive surgical techniques used are presented in Table 3. All 141 patients completed the TESS. Overall, 137 of the 141 patients completed the QLQ-C30 measure and 135 completed the 15D measure. Patients completed the measures postoperatively at a single point in time; the time from surgery to measurement ranged from 6 to 149 months (mean 62 months [SD 38]). The overall complication rate was 24% (34/141 patients), including 16% (23/141 patients) of major complications and 8% (11/141) of minor complications.

**Table 2:** Demographic and clinical data

	*n* = 141
Female, *n* (%)	72 (51)
Age, years, mean (SD)	65 (15)
BMI, mean (SD)	27 (5)
Tumor status (%)	
Primary	117 (83)
Recurrence	24 (17)
Tumor location, *n* (%)	
Proximal	110 (78)
Distal	31 (22)
Sarcoma subtype, *n* (%)	
Liposarcoma	56 (39.7)
Undifferentiated pleomorphic sarcoma	27 (19.1)
Sarcoma NOS	17 (12.1)
Leiomyosarcoma	16 (11.3)
Myxofibrosarcoma	9 (6.4)
Other	16 (11.4)
Tumor grade, *n* (%)	
Low	73 (51.8)
High	68 (48.2)
Tumor depth, with relation to deep fascia	
Superficial	40 (28.4)
Deep	101 (71.6)
Microscopical margins, *n* (%)	
Intralesional	12 (8.5)
Marginal	88 (62.4)
Wide	41 (29.1)
Tumor size, mean (SD), cm	8 (6)
Wound closure, *n* (%)	
Direct	89 (61.3)
Skin graft	22 (15.6)
Reconstruction	30 (21.3)
Radiotherapy, *n* (%)	
Preoperative	10 (7.1)
Postoperative	46 (32.6)
Chemotherapy, *n* (%)	22 (15.6)
Complications, *n* (%)	
Minor	11 (7.8)
Major	23 (16.3)
Time since surgery, months, mean (SD)	62 (38)

*n* number of patients;*SD* standard deviation

Location: proximal (groin, buttock, thigh) and distal (knee, lower leg, foot and ankle)

**Table 3:** Reconstructions (35 of 141 limb salvage patients)

	*n* = 35
Reconstruction	*n* (%)
Microvascular LD	5 (3.5)
Local fasciocutaneous flap	4 (2.8)
Microvascular ALT	4 (2.8)
Pedicled ALT	3 (2.1)
Microvascular gracilis flap	3 (2.1)
Gastrocnemicus muscle transposition	2 (1.4)
Pedicled TFL	2 (1.4)
Other*	12 (8.5)

*Others included three oncovascular reconstructions, as well as one each of pedicled sartorius, pedicled gracilis, pedicled suralis, microvascular TFL, microvascular scapula, microvascular radial forearm, propeller flaps, knee tumorprothesis and ligamentoplasty. One mirovascular LD and one pedicled gracilis included oncovascular reconstructions

*LD* Latissimus dorsi; *ALT* anterolateral thigh;* TFL* tensor faciae latae

### Functional Outcome

The mean score (range) for TESS, 15D mobility item score, and QLQ-C30 PF was 86 (12–100), 0.83 (0.47–1), and 75 (7–100), respectively. Depending on the used outcome measure, 19–51% of patients were completely asymptomatic; a maximum score was reported by 69 of 135 (51%) patients for the 15D mobility item, 26 of 135 (19%) patients for the QLQ-C30 PF scale, and 31 of 141 (22%) patients for the TESS.

The results of univariate and multivariate analyses are presented in Table 4, while non-significant univariate analysis results are presented in the electronic supplementary material. Multivariate linear regression analysis showed that lower functional outcome was statistically significantly associated with higher age, higher BMI, and the need for reconstructive surgery and radiotherapy. All three measures showed statistically significant results for age, two of three outcome measures (TESS and QLQ-C30 scale) showed statistically significant results for BMI and reconstructive surgery, and one of three outcome measures (TESS) showed statistically significant results for radiotherapy (Table 5). A sensitivity analysis excluding grade 1 liposarcomas is shown in Table 5. The same factors as in the main analysis were significantly associated with functional outcome, but the results of the multivariate analysis changed somewhat. High age and BMI treatment of recurrence and motor nerve, but not radiotherapy and reconstructive surgery, were significantly associated with functional outcome in the multivariate sensitivity analysis.

**Table 4:** Uni- and multivariate analysis of factors predictive for functional outcome in lower extremity STS patients

Characteristics	Univariate												Multivariate					
	TESS patients	TESS mean (SD)	*β*	*p* value	15D mob^1^ patients	15D mob^1^ mean (SD)	*β*	*p* value	PF^2^ patients	PF^2^ mean (SD)	*β*	*p* value	TESS *β*	*p* value	15D mob^1^ *β*	*p* value	PF^2^ *β*	*p* value
Eligible cases	141/141	86			135/141	83			135/141	75								
		(17)				(19)				(24)								
Age (years)^3^			− 0.3	< **0.01**			− 0.4	< **0.01**			− 0.6	< **0.01**	− 0.3	< **0.01**	− 0.4	< **0.01**	− 0.6	< **0.01**
18–40	10/10	98			09/10	97			9/10	96								
		(4)				(10)				(9)								
41–50	11/11	94			11/11	97			11/11	93								
		(8)				(9)				(8)								
51–60	19/19	86			18/19	80			19/19	74								
		(18)				(15)				(25)								
61–70	41/41	87			39/41	82			38/41	74								
		(16)				(19)				(23)								
71–80	38/38	87			37/38	84			36/38	79								
		(13)				(17)				(17)								
>80	22/22	75			21/22	73			22/22	55								
		(25)				(20)				(29)								
Obesity^3^			− 0.8	< **0.01**			− 0.5	0.11			− 0.9	**0.02**	− 0.6	**0.02**	− 0.4	0.26	− 0.9	**0.02**
No obesity	41/41	89			40/41	83			41/41	78								
		(14)				(19)				(24)								
Overwight	45/45	89			45/45	87			44/45	78								
		(16)				(18)				(22)								
Obesity	37/37	80			37/37	77			37/37	67								
		(17)				(19)				(24)								
Tumor status			− 5.2	**0.04**			− 11.9	**0.02**	112/117		− 10.7	**0.05**	− 3.5	0.37	− 3.1	0.51	0.2	0.98
Primary	117/117	88			111/117	85				77								
		(15)				(19)			23/24	(23)								
Recurrence	24/24	78			24/24	76				66								
		(23)				(18)				(26)								
Reconstruction			− 7.7	**0.02**			− 8.1	**0.03**			− 9.3	**0.05**	− 6.7	**0.04**	− 6.3	0.1	− 10.4	**0.03**
None	106/106	88			101/106	85			100/106	78								
		(16)				(19)				(22)								
Reconstruction	35/35	81			34/35	77			35/35	68								
		(19)				(18)				(26)								
Motor nerve resection			− 10.7	**0.03**			− 13.5	**0.01**			− 15.2	**0.03**	− 7.4	0.12	− 8.9	0.12	− 9.5	0.17
No	127/127	87			122/127	84			122/127	77								
		(15)				(18)				(22)								
Yes	14/14	77			13/14	71			13/14	62								
		(30)				(23)				(31)								
Tumor depth			− 5.1	0.11			− 9.2	**0.01**			− 8.6	0.06	− 0.2	0.95	− 5.1	0.17	− 2.7	0.55
Superficial	40/40	90			40/40	90			39/40	81								
		(17)				(17)				(23)								
Deep	101/101	85			95/101	80			96/101	73								
		(17)				(19)				(24)								
Margins			6.3	**0.01**			4.6	0.08			6.7	**0.05**	0.7	0.8	− 0.5	0.87	1.8	0.64
Intralesional	11/11	81			11-Nov	82			11-Nov	68								
		(17)				(18)				(23)								
Marginal	76/76	84			73/76	80			74/76	73								
		(19)				(19)				(26)								
Wide	54/54	91			51/54	87			50/54	80								
		(13)				(18)				(20)								
Radiotherapy			− 8.5	< **0.01**			− 6.3	**0.05**			− 5.8	0.16	− 7.5	**0.01**	− 4.9	0.17	− 4.5	0.3
No	83/83	90			80/83	86			78/83	78								
		(14)				(18)				(21)								
Yes	58/58	81			55/58	79			57/58	72								
		(20)				(20)				(27)								

Statistically significant univariate analysis results presented only (others reported in supplementary material)

*p* value < 0.05 were considered significant and are given in bold

*PF* physical function scale; * QoL* quality of life;* SD* standard deviation; *β*—unstandardized coefficients;*DC* direct closure

^1^15D mobility item. In order to improve comparability with the other measures the 15D scale of 0–1 is converted into 0–100

^2^QLQ-C30 PF scale

^3^Tested as continuous variable

Overweight and obesity was defined as BMI ≥ 25 kg/m^2^ and ≥ 30 kg/m^2^, respectively

Location: proximal (groin, buttock, thigh) and distal (knee, lower leg, foot and ankle)

Variables analyzed in univariate analysis: age, BMI, gender, sarcoma type, tumor grade, tumor status, surgery, location, depth, motoric nerve resection, reconstruction surgery, tumor size, radiotherapy, complications, follow-up time. Statistically insignificant results are presented as supplementary material

**Table 5:** Sensitivity analysis. Uni- and multivariate analysis of factors predictive for functional outcome in lower extremity STS patients (grade 1 liposarcomas (n = 31) excluded)

	Univariate												Multivariate					
Characteristics	TESS patients	TESS mean (SD)	*β*	*p* value	15D mob^1^ patients	15D mob^1^ mean (SD)	*β*	*p* value	PF^2^ patients	PF^2^ mean (SD)	*β*	*p* value	TESS *β*	*p* value	15D^1^ *β*	*p* value	PF^2^ *β*	*p* value
Eligible cases	110/110	86			104/110	83			105/110	75								
		(17)				(19)				(24)								
Age (years)^3^			− 0.3	< **0.01**			− 0.4	< **0.01**			− 0.5	< **0.01**	− 0.3	< **0.01**	− 0.3	< **0.01**	− 0.5	< **0.01**
18–40	8/8	97			7/8	96			7/8	95								
		(4)				(11)				(10)								
41–50	7/7	95			7/7	100			7/7	94								
		(7)				(0)				(8)								
51–60	19/19	86			18/19	80			19/19	74								
		(18)				(20)				(25)								
61–70	33/33	86			31/33	82			30/33	74								
		(16)				(19)				(23)								
71–80	28/28	86			27/28	82			27/28	78								
		(14)				(17)				(18)								
>80	15/15	74			14/15	75			15/15	57								
		(24)				(22)				(29)								
Obesity^3^			− 0.8	< **0.01**			− 0.7	0.07			− 1.4	< **0.01**	− 0.7	**0.01**	− 0.4	0.29	− 1.2	< **0.01**
No obesity	33/33	88			32/33	83			33/33	79								
		(17)				(18)				(23)								
Overwight	33/33	89			33/33	87			33/33	79								
		(16)				(18)				(23)								
Obesity	29/29	78			29/29	76			29/29	63								
		(18)				(20)				(25)								
Tumor status			− 18.3	< **0.01**			− 17.4	< **0.01**			− 20.5	< **0.01**	− 13.3	**0.02**	− 13.1	**0.05**	− 12.4	0.11
Primary	99/99	88			93/99	85			94/99	77								
		(16)				(19)				(23)								
Recurrence	11/11	69			11/11	67			11/11	57								
		(20)				(15)				(26)								
Reconstruction			− 7.9	**0.02**			− 8.7	**0.03**			− 10.4	**0.03**	− 5.4	0.1	− 5.3	0.18	− 8.2	0.09
None	75/75	88			70/75	86			70/75	79								
		(16)				(19)				(22)								
Reconstruction	35/35	81			34/35	77			35/35	68								
		(19)				(18)				(26)								
Motor nerve resection			− 7.8	0.15			− 17.1	< **0.01**			− 15.4	**0.05**	− 11.7	**0.03**	− 15.5	**0.02**	− 14.8	**0.05**
No	99/99	87			94/99	85			95/99	77								
		(16)				(18)				(23)								
Yes	11/11	79			10/11	67			10/11	61								
		(26)				(21)				61(30)								
Tumor depth			− 5.2	0.13			− 9.2	< **0.01**			− 8.6	0.09	1.8	0.6	− 4	0.33	0	0.99
Superficial	37/37	89			37/37	90			36/37	81								
		(17)				(17)				(24)								
Deep	73/73	84			67/73	79			69/73	72								
		(17)				(19)				(23)								
Margins			8.6	< **0.01**			7.7	**0.02**			8.8	**0.03**	2.9	0.38	1.1	0.78	3.7	0.43
Intralesional	06/6	73			06/6	77			06/6	62								
		(18)				(20)				(27)								
Marginal	64/64	84			60/64	80			62/64	73								
		(18)				(20)				(26)								
Wide	40/40	92			38/40	89			37/40	81								
		(13)				(16)				(18)								
Radiotherapy			− 10.2	< **0.01**			− 6.2	0.1			− 8.8	0.06	− 5.2	0.16	− 2.5	0.57	− 3.1	0.56
No	55/55	91			52/55	86			51/55	80								
		(12)				(18)				(18)								
Yes	55/55	81			52/55	80			54/55	71								
		(20)				(20)				(28)								

Statistically significant univariate analysis results presented only (others reported in supplementary material)

*p* value < 0.05 were considered significant and are given in bold

*PF* physical function scale;* QoL* quality of life;*SD* standard deviation; *β*—unstandardized coefficients;* DC* direct closure

^1^15D mobility item. In order to improve comparability with the other measures the 15D scale of 0–1 is converted into 0–100

^2^QLQ-C30 PF scale

^3^Tested as continuous variable

Overweight and obesity was defined as BMI ≥ 25 kg/m^2^ and ≥ 30 kg/m^2^, respectively

Location: proximal (groin, buttock, thigh) and distal (knee, lower leg, foot and ankle)

Variables analyzed in univariate analysis: age, BMI, gender, sarcoma type, tumor grade, tumor status, surgery, location, depth, motoric nerve resection, reconstruction surgery, tumor size, radiotherapy, complications, follow-up time

### Health-Related Quality of Life

The mean score (range) for the 15D and QLQ-C30 QoL was 0.88 (0.44–1) and 73 (0–100), respectively. Based on the HRQoL measures, 13–14% of patients had unimpaired HRQoL. A maximum score was reported by 19 of 135 (14%) patients for the 15D overall score and 18 of 137 (13%) patients for the QLQ-C30 QoL scale.

The results of univariate and multivariate analyses are presented in Table 6, while non-significant univariate analysis results are presented in the electronic supplementary material. Multivariate linear regression analysis showed that lower HRQoL was statistically significantly associated with higher age, higher BMI, and the need for reconstructive surgery. Both measures showed statistically significant results for age and BMI, and one of two (QLQ-C30 QoL scale) showed statistically significant results for the need for reconstructive surgery (Table 6). The results of the sensitivity analysis excluding grade 1 liposarcomas was similar to the main analysis (electronic supplementary material).

**Table 6:** Uni- and multivariate analysis of factors predictive for HRQoL in lower extremity STS patients

Characteristics	Univariate								Multivariate			
	15D patients	15D^1^ mean (SD)	*β*	*p* value	QLQ-C30 patients	QoL^2^mean (SD)	*β*	*p* Value	15D^1^ *β*	*p* value	QoL^2^ *β*	*p* value
Eligible cases	135/141	88			137/141	73						
		(11)				(22)						
Age (years)^3^			− 0.2	< **0.01**			− 0.4	< **0.01**	− 0.2	< **0.01**	− 0.3	< **0.01**
18–40	9/10	96			9/10	88						
		(6)				(10)						
41–50	11/11	94			11/10	82						
		(6)				(15)						
51–60	18/19	87			19/19	71						
		(15)				(24)						
61–70	39/41	87			39/41	74						
		(11)				(22)						
71–80	37/38	90			37/38	76						
		(9)				(17)						
>80	21/22	81			22/22	57						
		(13)				(25)						
Obesity^3^			− 0.5	**0.01**			− 0.7	**0.04**	− 0.5	< **0.01**	− 0.7	**0.05**
No obesity	40/41	90			41/41	76						
		(10)				(18)						
Overwight	45/45	89			45/45	77						
		(10)				(20)						
Obesity	37/37	84			37/37	67						
		(12)				(24)						
Tumor status			− 5.2	**0.04**			− 11.9	**0.02**	0.1	0.96	− 3.7	0.45
Primary	111/117	89			113/117	75						
		(11)				(21)						
Recurrence	24/24	84			23/24	63						
		(12)				(25)						
Reconstruction			− 3.4	0.14			− 9.5	**0.03**	− 3.7	0.09	− 11.1	< **0.01**
None	101/106	89			102/106	75						
		(11)				(21)						
Reconstruction	34/35	85			35/35	66						
		(12)				(24)						

Statistically significant univariate analysis results presented only (others reported in supplementary material)

*p* value < 0.05 were considered significant and are given in bold

*PF* physical function factor;* QoL* quality of life;* SD* standard deviation; *β* unstandardized coefficients

^1^15D overall score. In order to improve comparability with the other measures the 15D scale of 0–1 is converted into 0–100

^2^QLQ-C30 QoL scale

^3^Tested as continuous variable

Overweight and obesity was defined as BMI ≥ 25 kg/m^2^ and ≥ 30 kg/m^2^, respectively

Variables analyzed in univariate analysis: age, BMI, gender, sarcoma type, tumor grade, tumor status, surgery, location, depth, motoric nerve resection, reconstruction surgery, tumor size, radiotherapy, complications, follow-up time

## Discussion

Functional and HRQoL outcomes are important aspects in sarcoma treatment. This study used a large sample of patients with lower extremity STS and focused on postoperative functional outcome and HRQoL. Mean functional outcome and HRQoL were generally good, with a considerable proportion having unimpaired functional outcome and HRQoL. We found that both outcomes were most consistently related to the patient-related factors of age and BMI, while the treatment-related factors of reconstructive surgery and radiotherapy also had an effect, especially on functional outcome.

### Functional Outcome

Different measures measure different aspects of functional outcome. The TESS measures only lower limb activity in relation to activity limitations,^9^–^13^ the QLQ-C30 PF scale measures certain physical abilities not exclusively related to lower limb function,^14^ and the 15D mobility item measures the need for assistance or how much help a patient needs in daily life. The TESS measure has 30 questions, each having five different possible answers, whereas the 15D mobility item has only one question and five possible answers. The 15D mobility item is one of the 15 items included in the overall 15D HRQoL score, which may explain the different proportions of completely asymptomatic patients (19–51%) measured by these three measures. The TESS is the primary functional outcome measure in patients with lower extremity STS, since it specifically measures the function affected by the disease and its treatment sequelae, but, in part, the same factors also affect the other two measures, which measure overall physical performance.

Previously published studies regarding functional outcome after sarcoma surgery include varying numbers of patients with lower extremity STS, and the heterogeneity also varies. This makes comparison of functional outcome measure scores between different studies difficult. In a large literature review that focused on patients with lower limb STS, the mean postoperative TESS score was 83.3.^3^ In our study, the mean TESS score was similar, i.e. 86.4.

Only a few previous studies have analyzed how patient-related factors affect functional outcome in patients with lower extremity STS.^7^^,^^8^ We found that higher age and higher BMI were the most consistent determinants of functional outcome in patients with lower limb STS, and our results are supported by the studies of Davis et al., which include only patients with lower extremity^7^ or both upper and lower extremity STS.^24^ Heaver et al. also reported that higher age reduced functional outcome in a heterogenic population of extremity tumor patients.^25^ Banierink et al. found that elderly patients had significantly decreased functional outcome compared with younger patients after a pelvic ring injury, due to age-related vulnerability and limited rehabilitation capacity in elderly patients.^26^ Houdek et al. reported the opposite result for BMI in two different studies of patients with upper or lower limb STS,^8^^,^^27^ whereas no difference in the mean TESS score was found between obese and non-obese patients. However, the study of a cohort of patients by Heaver et al., which included bone, hematological, benign aggressive tumors, and metastatic disease, reported that higher BMI was associated with reduced functional outcome.^25^

Some studies have found that female sex is related to inferior postoperative functional outcome in patients with extremity tumor.^4^^,^^25^ These studies have included either bone and benign aggressive tumors or upper extremity sarcomas. In the present analysis, which included only patients with lower extremity STS, sex did not affect postoperative functional outcomes. Our results are supported by the study by Davis et al., which also only included patients with lower extremity STS.^7^

In the present analysis, tumor depth, a disease-specific factor, was significantly related to outcome in univariate analysis only. Again, a similar result was reported by Davis et al.,^7^ who also found that depth was a significant factor in univariate but not multivariate analysis. On the other hand, Saebye and colleagues found that deep tumors were statistically significantly associated with decreased functional outcome in patients with extremity STS, including upper extremities.^4^ They also reported that patients with lower extremity STS are at higher risk of functional impairment compared with patients with upper extremity STS, which is supported by the study of Weschenfelder et al.^28^ Saebye et al. included upper extremities in their tumor depth analysis, which might make an impact. Gerrand et al. found no significant impairment in TESS score in superficial tumors (mean 86.4% preoperatively vs. 90.9% postoperatively), but worse outcome in deep tumors (mean TESS 83.0% preoperatively vs. 79.4% postoperatively).^29^ The difference in outcome is not necessarily of practical importance as the minimal clinically important difference has been defined as ≥ 4–10 for the TESS.^17^ On the other hand, the difference in postoperative TESS score between superficial and deep tumors in the study by Gerrand et al. exceeded this threshold (90.9 vs. 79.4).^29^ Interestingly, the TESS score was higher postoperatively in superficial tumors and lower in deep tumors.^29^

In addition to patient- and tumor-related factors, the treatment-related factors of radiotherapy and reconstructive surgery were associated with worse functional outcome in the present study. Similar results were seen in the study by Götzl et al.,^30^ who studied patients with both upper and lower limb STS and found that patients treated with neoadjuvant radiotherapy had lower functional outcome compared with patients without radiotherapy.^30^ Lower functional outcome scores in patients treated with radiotherapy have also been reported in other studies.^4^^,^^31^ Davis et al. reported that radiotherapy was associated with lower functional outcome in univariate analysis but not in multivariate analysis,^7^ whereas Townley et al. found no difference in functional outcome between extremity sarcoma patients receiving or not receiving preoperative irradiation;^32^ however, Townley et al. also included bone sarcomas in the control group and functional outcome was assessed in 21 of 40 extremity patients. Furthermore, Townley et al. reported that the TESS scores for the pre-irradiation and control groups were 81.7 (*n* = 15) and 92.4 (*n* = 6), respectively. In their study including patients with trunk, lower and upper extremity, amputation, and bone sarcoma patients, Weschenfelder et al. found a significant association between adjuvant radiotherapy and lower functional outcome.^28^

In our study, reconstructive surgery was associated with lower functional outcome. Similar results were published by Kang et al., where the flap reconstruction group, especially the free-flap group, had lower functional outcome compared with patients with primary wound closure.^33^ Like radiotherapy, reconstructive surgery is often needed in cases with more severe disease, usually in patients with deep and large tumors.

Postoperative complications were associated with somewhat worse functional outcomes in our study, but not statistically significantly. Davis et al. found that complications were associated with lower functional outcome, as measured by the MSTS 87 and 93 measures but not by the TESS measure.^7^ Two studies, by Pradhan et al. and Stoeckle et al., concluded that surgical complications led to decreased functional outcome. The former study included adductor compartment sarcomas only, with a wound complication rate of 36%, while the latter study included patients with trunk STS as well as both upper and lower limb STS.^34^^,^^35^ In addition, Davis et al. reported that complications were related to worse functional outcome in patients with upper or lower extremity STS.^24^ Interestingly, Slump et al. reported that complications were associated with worse functional outcome in patients with upper extremity STS but not in patients with lower extremity STS.^36^ These findings suggest that patients with upper and lower extremity STS should be analyzed separately for functional outcome.

We performed a sensitivity analysis excluding cases with grade 1 liposarcomas, since these cases are partly managed according to different surgical principles and radiotherapy is not recommended in these cases. The univariate sensitivity analysis of functional outcome gave the same results as in the main analysis, while radiotherapy and reconstructive surgery lost their significance in the multivariate analysis. On the other hand, motor nerve resection and surgical treatment of a local recurrence rose to significance in the multivariate sensitivity analysis; however, patient age and BMI were still the strongest determinants of functional outcome in the multivariate analysis. The somewhat different results in the main and sensitivity analyses may be partly due to the fact that many treatment-related factors are closely related to each other, which may render a multivariate statistical model unstable. The sensitivity analysis of HRQoL did not differ substantially from the main analysis.

### Health-Related Quality of Life

Only a few previous studies have focused on HRQoL in patients with exclusively lower extremity STS,^5^^,^^7^ and even fewer studies have focused on long-term outcome. Overall, the mean QLQ-C30 QoL result in our study is comparable with previously published results in patients with extremity sarcoma, where QLQ-C30 QoL scores ranging from 65.1 to 76.6 have been reported.^5^^,^^37^^,^^38^ These scores are similar to that of the general population, in which the normative mean scores for the QLQ-C30 QoL measure in 13 European countries, Canada, and the Unites States have ranged from 62.6 to 71.1.

Although we found the HRQoL of sarcoma patients to be close to the expected level of the general population, the treatment of sarcoma may affect HRQoL, at least after the short-term follow-up.^24^^,^^39^^,^^40^ In our study, we found that the only predictive factors statistically significantly influencing postoperative long-term HRQoL were BMI, age, and the need for reconstructive surgery. Both the 15D and QLQ-C30 QoL measures demonstrated that higher age and higher BMI decrease HRQoL, and only the QLQ-C30 QoL measure indicated that reconstructive surgery, or the need for it, negatively influenced HRQoL.

Previous studies generally support our findings relating age to HRQoL but results on the association between treatment-related factors and HRQoL vary. Furthermore, we have not identified any previous studies associating BMI with HRQoL in patients with lower extremity STS. Davis et al. reported that age is a significant factor affecting postoperative HRQoL in patients with lower extremity STS, based on the SF-36 HRQoL measure,^7^ while Davidson et al. reported that higher age and female sex decrease HRQoL, but found no association between HRQoL and flap coverage, use of radiotherapy, or wound complications at 1 year postoperatively.^6^ The study by Davidson et al. included patients with upper and lower extremity STS, as well as patients with amputations.^6^ However, Götzl et al. studied patients with both upper and lower limb STS and found that patients treated with neoadjuvant radiotherapy had lower HRQoL compared with patients who were not treated with radiotherapy.^30^ The study by Weschenfelder et al., which included patients with STS in the trunk and extremity, as well as bone sarcomas, did not find any impact of radiotherapy on HRQoL.^28^ Saebye et al. reported that only female sex was associated with the decreased postoperative QLQ-C30 score in patients with extremity STS. In their analysis, Saebye et al. included both upper and lower extremities.

Our study has some limitations, including those inherent to its retrospective design, as well as the absence of preoperative functional outcome and HRQoL data. However, a strength of this study was its large sample size and homogeneity, including patients with lower extremity STS from a large tertiary academic referral center who were treated with limb-salvage surgery. The participation rate was 65%, which is relatively high for a mail survey. Functional and HRQoL scores were prospectively and structurally assessed using well-validated tools.

## Conclusion

Post-treatment functional outcome of patients with lower extremity STS is affected by BMI, age, and the need for reconstructive surgery and radiotherapy. The most significant predictors that affect HRQoL are BMI, age, and the need for reconstructive surgery. Although tumor- and treatment-related factors had an impact, patient-related factors such as age and BMI were the most consistent and significant determinants of both functional outcome and HRQoL.

## Supplementary Information

Supplementary material 1 (DOCX 43 kb)

Supplementary material 2 (DOCX 36 kb)

Supplementary material 3 (DOCX 25 kb)

## Data Statement

All data are available from the corresponding author upon reasonable request.
