# Supplementary material for: Soft Tissue Sarcoma of Lower Extremity: Functional Outcome and Quality of Life
Source: Ann Surg Oncol. 2021 Mar 19;28(11):6892–905. doi: 10.1245/s10434-021-09774-6 (PMC8460521; doi:10.1245/s10434-021-09774-6)
Supplement: Supplementary file 1 — Supplementary material 1 (DOCX 43 kb) [file 10434_2021_9774_MOESM1_ESM.docx]

Supplemental material. Supplemental table 1. Association between demographic and clinical variables and functional outcome in lower extremity STS patients.

|  |  |  |  |  |  |  |  |  |  |  |  |  |
| --- | --- | --- | --- | --- | --- | --- | --- | --- | --- | --- | --- | --- |
| **Characteristics** | **TESS**  **patients** | **TESS**  **mean (SD)** | **β** | **p-**  **value** | **15D mob***  **patients** | **15D mob^1^**  **mean**  **(SD)** | **β** | **p-**  **value** | **PF****  **patients** | **PF^2^**  **mean**  **(SD)** | **β** | **p-value** |
| **Eligible cases** | 141/141 | 86  (17) |  |  | 135/141 | 83  (19) |  |  | 135/141 | 75  (24) |  |  |
| **Gender**  Female  Male | 72/72  69/69 | 85  (18)  88  (16) | 3.2 | 0.27 | 71/72  64/69 | 83  (19)  83  (19) | 0.7 | 0.84 | 70/71  65/69 | 73  (24)  78  (23) | 4.8 | 0.24 |
| **Age (years)^3^**  18-40  41-50  51-60  61-70  71-80  >80 | 10/10  11/11  19/19  41/41  38/38  22/22 | 98  (4)  94  (8)  86  (18)  87  (16)  87  (13)  75  (25) | -0.3 | **<0.01** | 9/10  11/11  18/19  39/41  37/38  21/22 | 97  (10)  97  (9)  80  (15)  82  (19)  84  (17)  73  (20) | -0.4 | **<0.01** | 9/10  11/11  19/19  38/41  36/38  22/22 | 96  (9)  93  (8)  74  (25)  74  (23)  79  (17)  55  (29) | -0.6 | **<0.01** |
| **Follow-up time (years)^3^**  ≥2  2 - ≥5  5- ≥ 9  >9 | 23/23  51/51  50/50  17/17 | 83  (19)  87  (17)  89  (16)  85  (18) | 0.0 | 0.75 | 21/23  49/51  50/50  17/17 | 71  (28)  77  (22)  77  (24)  69  (24) | 0.0 | 0.95 | 21/23  49/51  48/50  17/17 | 79  (21)  84  (19)  85  (17)  79  (20) | 0.0 | 0.71 |
| **Obesity^3^**  No obesity  Overwight  Obesity | 41/41  45/45  37/37 | 89  (14)  89  (16)  80  (17) | -0.8 | **<0.01** | 40/41  45/45  37/37 | 83  (19)  87  (18)  77  (19) | -0.5 | 0.11 | 41/41  44/45  37/37 | 78  (24)  78  (22)  67  (24) | -0.9 | **0.02** |
| **Location**  Proximal  Distal | 104/110  31/31 | 87  (18)  86  (16) | -0.9 | 0.81 | 104/110  31/31 | 83  (19)  84  (18) | 0.9 | 0.82 | 104/110  31/31 | 76  (23)  72  (25) | -4.2 | 0.39 |
| **Tumor status**  Primary  Recurrence | 117/117  24/24 | 88  (15)  78  (23) | -5.2 | **0.04** | 111/117  24/24 | 85  (19)  76  (18) | -11.9 | **0.02** | 112/117  23/24 | 77  (23)  66  (26) | -10.7 | **0.05** |
| **Sarcoma subtype**  Liposarcoma  UPS  Sarcoma NOS  LMS  MFS  Others | 56/56  27/27  17/17  16/16  9/9  16/16 | 90  (15)  84  (17)  85  (23)  84  (17)  85  (15)  84  (21) | -1.1 | 0.20 | 53/56  26/27  16/17  16/16  9/9  15/16 | 86  (18)  79  (19)  84  (20)  79  (21)  81  (19)  85  (18) | -0.4 | 0.64 | 52/56  27/27  17/17  15/16  9/9  15/16 | 78  (21)  69  (22)  78  (31)  68  (28)  77  (23)  80  (22) | 0.0 | 0.98 |
| **Grade**  Low  High | 73/73  68/68 | 88  (17)  84  (17) | -3.7 | 0.20 | 71/73  64/68 | 86  (19)  80  (18) | -5.2 | 0.11 | 69/73  66/68 | 77  (24)  74  (23) | -3.0 | 0.47 |
| **Tumor size^3^ (cm)**  1-3  >3 - 6  >6 - 10  >10 – 15  >15 | 27/27  39/39  40/40  19/19  14/14 | 86  (16)  87  (17)  85  (19)  83  (21)  91  (9) | 0.0 | 0.60 | 27/27  37/39  38/40  17/19  14/14 | 84  (18)  85  (18)  76  (23)  76  (23)  86  (14) | 0.0 | 0.72 | 26/27  36/39  39/40  18/19  14/14 | 74  (26)  76  (24)  77  (22)  68  (28)  77  (18) | 0.0 | 0.99 |
| **Operation**  Excision  Myectomy | 103/103  38/38 | 87  (17)  85  (18) | -0.8 | 0.64 | 101/103  34/38 | 85  (18)  78  (20) | -3.4 | 0.07 | 100/103  35/38 | 75  (24)  75  (23) | -0.1 | 0.95 |
| **Reconstruction**  None  Reconstruction | 106/106  35/35 | 88  (16)  81  (19) | -7.7 | **0.02** | 101/106  34/35 | 85  (19)  77  (18) | -8.1 | **0.03** | 100/106  35/35 | 78  (22)  68  (26) | -9.3 | **0.05** |
| **Motor nerve resection**  No  Yes | 127/127  14/14 | 87  (15)  77  (30) | -10.7 | **0.03** | 122/127  13/14 | 84  (18)  71  (23) | -13.5 | **0.01** | 122/127  13/14 | 77  (22)  62  (31) | -15.2 | **0.03** |
| **Tumor depth**  Superficial  Deep | 40/40  101/101 | 90  (17)  85  (17) | -5.1 | 0.11 | 40/40  95/101 | 90  (17)  80  (19) | -9.2 | **0.01** | 39/40  96/101 | 81  (23)  73  (24) | -8.6 | **0.06** |
| **Margins**  Intralesional  Marginal  Wide | 11/11  76/76  54/54 | 81  (17)  84  (19)  91  (13) | 6.3 | **0.01** | 11/11  73/76  51/54 | 82  (18)  80  (19)  87  (18) | 4.6 | 0.08 | 11/11  74/76  50/54 | 68  (23)  73  (26)  80  (20) | 6.7 | **0.05** |
| **Radiotherapy**  No  Yes | 83/83  58/58 | 90  (14)  81  (20) | -8.5 | **<0.01** | 80/83  55/58 | 86  (18)  79  (20) | -6.3 | **0.05** | 78/83  57/58 | 78  (21)  72  (27) | -5.8 | 0.16 |
| **Chemotherapy**  None  Yes | 119/119  22/22 | 87  (18)  85  (15) | -1.2 | 0.76 | 115/119  20/22 | 84  (19)  80  (18) | -3.2 | 0.48 | 113/119  22/22 | 75  (24)  75  (22) | -0.1 | 0.98 |
| **Complications**  None  Minor  Major | 107/107  11/11  23/23 | 88  (17)  83  (22)  82  (16) | -2.8 | 0.15 | 102/107  10/11  23/23 | 84  (19)  78  (21)  80  (17) | -2.2 | 0.30 | 102/107  10/11  23/23 | 76  (23)  72  (33)  72  (21) | -2.2 | 0.41 |

LMS – Leiomyosarcoma; MFS – Myxofibrosarcoma; PF - Pedicled flap; MF - Micovascular flap

PF – physical function scale; QoL – quality of life; DC- direct closure; STSG - split-thickness skin graft.

^1^ 15D mobility item. In order to improve comparability with the other measures the 15D scale of 0-1 is converted into 0-100

^2^ QLQ-C30 PF scale

^3^ Tested as continuous variable

Overweight and obesity was defined as BMI ≥25 kg/m2 and ≥30 kg/m2, respectively.

Location: proximal (groin, buttock, thigh) and distal (knee, lower leg, foot and ankle).
