# Supplementary material for: Soft Tissue Sarcoma of Lower Extremity: Functional Outcome and Quality of Life
Source: Ann Surg Oncol. 2021 Mar 19;28(11):6892–905. doi: 10.1245/s10434-021-09774-6 (PMC8460521; doi:10.1245/s10434-021-09774-6)
Supplement: Supplementary file 2 — Supplementary material 2 (DOCX 36 kb) [file 10434_2021_9774_MOESM2_ESM.docx]

Supplemental material. Supplemental table 2. Association between demographic and clinical variables and health related quality of life outcome in lower extremity STS patients.

|  |  |  |  |  |  |  |  |  |
| --- | --- | --- | --- | --- | --- | --- | --- | --- |
| **Characteristics** | **15D**  **patients** | **15D^1^**  **mean**  **(SD)** | **β** | **p-**  **value** | **QLQ-C30**  **patients** | **QoL^2^**  **mean**  **(SD)** | **β** | **p-**  **value** |
| **Eligible cases** | 135/141 | 88  (11) |  |  | 137/141 | 73  (22) |  |  |
| **Gender**  Female  Male | 71/72  64/69 | 88  (12)  89  (11) | 1.0 | 0.62 | 72/72  65/69 | 72  (23)  74  (20) | 2.1 | 0.58 |
| **Age^3^ (years)**  18-40  41-50  51-60  61-70  71-80  >80 | 9/10  11/11  18/19  39/41  37/38  21/22 | 96  (6)  94  (6)  87  (15)  87  (11)  90  (9)  81  (13) | -0.2 | **<0.01** | 9/10  11/11  19/19  39/41  37/38  22/22 | 88  (10)  82  (15)  71  (24)  74  (22)  76  (17)  57  (25) | -0.4 | **<0.01** |
| **Follow-up time^3^ (years)**  ≥2  2 - ≥5  5- ≥ 9  >9 | 21/23  49/51  48/50  17/17 | 84  (16)  89  (9)  89  (11)  88  (12) | 0.0 | 0.52 | 21/23  49/51  50/50  17/17 | 68  (27)  74  (17)  74  (23)  72  (24) | 0.0 | 0.63 |
| **Obesity^3^**  No obesity  Overwight  Obesity | 40/41  45/45  37/37 | 90  (10)  89  (10)  84  (12) | -0.5 | **0.01** | 41/41  45/45  37/37 | 76  (18)  77  (20)  66  (24) | -0.7 | **0.04** |
| **Location**  Proximal  Distal | 104/110  31/31 | 89  (10)  85  (14) | -3.4 | 0.15 | 106/110  31/31 | 75  (20)  67  (26) | -7.1 | 0.11 |
| **Tumor status**  Primary  Recurrence | 111/117  24/24 | 89  (11)  84  (12) | -5.2 | **0.04** | 113/117  24/24 | 75  (21)  63  (25) | -11.9 | **0.02** |
| **Sarcoma subtype**  Liposarcoma  UPS  Sarcoma NOS  LMS  MFS  Others | 53/56  26/27  16/17  16/16  9/9  15/16 | 88  (12)  87  (9)  88  (13)  86  (12)  90  (8)  90  (13) | 0.1 | 0.80 | 53/56  27/27  17/17  16/16  9/9  15/16 | 72  (22)  74  (19)  75  (25)  69  (27)  75  (12)  73  (23) | 0.1 | 0.94 |
| **Grade**  Low  High | 71/73  64/68 | 89  (13)  87  (10) | -1.7 | 0.39 | 71/73  66/68 | 73  (22)  72  (22) | -1.0 | 0.79 |
| **Tumor size^3^ (cm)**  1-3  >3 - 6  >6 - 10  >10 – 15  >15 | 27/27  37/39  38/40  17/19  14/14 | 87  (12)  90  (10)  86  (12)  86  (14)  93  (6) | 0.0 | 0.29 | 27/27  37/39  39/40  18/19  14/14 | 73  (20)  72  (21)  72  (25)  72  (26)  79  (14) | 0.0 | 0.51 |
| **Operation**  Excision  Myectomy | 101/103  34/38 | 88  (11)  87  (12) | -0.4 | 0.70 | 102/103  35/38 | 72  (21)  75  (24) | 1.0 | 0.63 |
| **Reconstruction**  None  Reconstruction | 101/106  34/35 | 89  (11)  85  (12) | -3.4 | 0.14 | 102/106  35/35 | 75  (21)  66  (24) | -9.5 | **0.03** |
| **Motor nerve resection**  No  Yes | 122/127  13/14 | 88  (11)  83  (15) | -5.2 | 0.12 | 124/127  13/14 | 74  (21)  64  (30) | -9.8 | 0.13 |
| **Tumor depth**  Superficial  Deep | 40/40  95/101 | 90  (12)  87  (11) | -3.5 | 0.10 | 40/40  97/101 | 77  (18)  71  (23) | -5.8 | 0.16 |
| **Margins**  Intralesional  Marginal  Wide | 11/11  73/76  51/54 | 89  (8)  87  (12)  90  (12) | 1.5 | 0.35 | 11/11  75/76  51/54 | 69  (19)  72  (23)  76  (21) | 3.6 | 0.25 |
| **Radiotherapy**  No  Yes | 80/83  55/58 | 89  (11)  87  (12) | -2.1 | 0.29 | 80/83  57/58 | 74  (21)  72  (22) | -2.1 | 0.59 |
| **Chemotherapy**  None  Yes | 115/119  20/22 | 88  (12)  88  (9) | 0.0 | 0.99 | 115/119  22/22 | 72  (22)  80  (19) | 7.8 | 0.13 |
| **Complications**  None  Minor  Major | 102/107  10/11  23/23 | 88  (11)  87  (18)  89  (9) | 0.3 | 0.79 | 104/107  10/11  23/23 | 75  (21)  68  (32)  68  (20) | -3.6 | 0.14 |

LMS – Leiomyosarcoma; MFS – Myxofibrosarcoma; PF - Pedicled flap; MF - Micovascular flap

PF – physical function scale; QoL – quality of life; DC- direct closure; STSG - split-thickness skin graft.

^1^ 15D overall score. In order to improve comparability with the other measures the 15D scale of 0-1 is converted into 0-100

^2^ QLQ-C30 QoL scale

^3^ Tested as continuous variable

Overweight and obesity was defined as BMI ≥25 kg/m2 and ≥30 kg/m2, respectively.

Location: proximal (groin, buttock, thigh) and distal (knee, lower leg, foot and ankle).
