# Supplementary material for: Soft Tissue Sarcoma of Lower Extremity: Functional Outcome and Quality of Life
Source: Ann Surg Oncol. 2021 Mar 19;28(11):6892–905. doi: 10.1245/s10434-021-09774-6 (PMC8460521; doi:10.1245/s10434-021-09774-6)
Supplement: Supplementary file 3 — Supplementary material 3 (DOCX 25 kb) [file 10434_2021_9774_MOESM3_ESM.docx]

Supplemental material. Supplemental table 3 ~~5~~. Sensitivity analysis. Uni- and multivariate analysis of factors predictive for HRQoL in lower extremity STS patients.

|  | **UNIVARIATE** | | | | | | | | **MULTIVARIATE** | | | |
| --- | --- | --- | --- | --- | --- | --- | --- | --- | --- | --- | --- | --- |
| **Characteristics** | **15D**  **patients** | **15D^1^**  **mean**  **(SD)** | **β** | **p-**  **value** | **QLQ-C30**  **patients** | **QoL^2^**  **mean**  **(SD)** | **β** | **p-**  **value** | **15D^1^**  **β** | **p-**  **value** | **QoL^2^**  **β** | **p-**  **value** |
| **Eligible cases** | 104/110 | 88  (11) |  |  | 106/110 | 73  (22) |  |  |  |  |  |  |
| **Age (years)^3^**  18-40  41-50  51-60  61-70  71-80  >80 | 7/8  7/7  18/19  31/33  27/28  14/15 | 98  (2)  94  (8)  87  (15)  87  (10)  90  (8)  80  (12) | -0.2 | **<0.01** | 7/8  7/7  19/19  31/33  27/28  15/15 | 88  (9)  81  (20)  71  (24)  74  (21)  75  (18)  58  (27) | -0.4 | **0.02** | -0.2 | **<0.01** | -0.3 | **0.03** |
| **Obesity^3^**  No obesity  Overwight  Obesity | 32/33  33/33  29/29 | 89  (10)  90  (9)  82  (13) | -0.6 | **<0.01** | 33/33  33/33  29/29 | 76  (17)  78  (20)  61  (25) | -1.1 | **<0.01** | -0.6 | **<0.01** | -0.9 | **0.02** |
| **Tumor status**  Primary  Recurrence | 93/99  11/11 | 89  (11)  80  (11) | -17.4 | **<0.01** | 95/99  11/11 | 75  (21)  53  (23) | -22.2 | **<0.01** | -2.9 | 0.44 | -13.8 | 0.06 |
| **Reconstruction**  None  Reconstruction | 70/75  34/35 | 89  (11)  85  (12) | -3.6 | 0.13 | 71/75  35/35 | 76  (20)  66  (24) | -10.5 | **0.02** | -2.5 | 0.29 | -8.6 | **0.05** |

Statistically significant univariate analysis results presented only (others reported in supplementary material).

PF – physical function factor; QoL – quality of life; SD – standard deviation; β – unstandardized coefficients.

^1^ 15D overall score. In order to improve comparability with the other measures the 15D scale of 0-1 is converted into 0-100

^2^ QLQ-C30 QoL scale

^3^ tested as continuous variable

Overweight and obesity was defined as BMI ≥25 kg/m^2^ and ≥30 kg/m^2^, respectively.

Variables analyzed in univariate analysis: age, BMI, gender, sarcoma type, tumor grade, tumor status, surgery, location, depth, motoric nerve resection, reconstruction surgery, tumor size, radiotherapy, complications, follow-up time.
